# Causative organisms of urinary tract infections and their drug sensitivity: an analysis from various aspects

**DOI:** 10.3389/fpubh.2025.1487721

**Published:** 2025-10-29

**Authors:** Xinwei Li, Yuanpeng Zhang, Wen Xiao, Xiaoping Zhang, Lei Liu

**Affiliations:** ^1^Department of Urology, Union Hospital, Tongji Medical College, Huazhong University of Science and Technology, Wuhan, China; ^2^Institute of Urology, Union Hospital, Tongji Medical College, Huazhong University of Science and Technology, Wuhan, China; ^3^Shenzhen Huazhong University of Science and Technology Research Institute, Shenzhen, China

**Keywords:** causative organism, urinary tract infection, drug susceptibility, drug resistance, antibiotics

## Abstract

**Objective:**

Urinary tract infection is a prevalent and complex clinical condition. To treat urinary tract infections more effectively, we sought to describe the distribution and antibiotic susceptibility of causative organisms in patients.

**Materials and methods:**

We retrospectively analyzed 3,685 patients with urinary tract infections between 2022 and 2023, treated at the Department of Urology of Wuhan Union Hospital. Clinical data, urine culture results, and drug sensitivity test data were collected for further analysis.

**Results:**

Of all 3,685 patients with positive urine cultures, 3,899 strains of causative organisms were isolated. Gram-negative bacteria (2,242/3899, 57.50%) were the most common causative organisms, among which *Escherichia coli* (1,250/3899, 32.06%) was the most common species. Drug sensitivity tests showed that most pathogens exhibited high sensitivity to restricted antibiotics (e.g., carbapenems) and cephalosporins (nearly 100 and 90%, respectively), but high resistance rates to quinolones and macrolides (over 50%). Comparing the distribution of causative organisms and drug sensitivity between 2022 and 2023, we found that the proportion of *E. coli* and *Proteus mirabilis* have increased significantly (*p* = 0.0066 and *p* = 0.0003, respectively), while the proportion of *Enterococcus faecium* have decreased significantly (*p* = 0.0419). Compared to non-stone infections, stone-associated infections showed a significantly higher proportion of *P. mirabilis* (*p* < 0.0001), consistent with its role in magnesium ammonium phosphate stones formation. Significant differences in pathogen distribution were also observed between outpatient and ward settings (*E. coli*, *Enterococcus faecalis*, *Klebsiella pneumoniae*, *Staphylococcus epidermidis*, *Enterobacter aerogenes*, *E. faecium*, *Pseudomonas aeruginosa*, and *Acinetobacter baumannii*).

**Conclusion:**

Based on the above results, cephalosporins are recommended to be applied in the initial empirical treatment, with timely adjustment according to the results of antimicrobial susceptibility testing.

## 1 Introduction

Urinary tract infection is defined as symptomatic bacterial colonization of the urinary tract. It is one of the most common infectious diseases worldwide, second only to upper respiratory tract infections (1). The latest statistics indicate that in the United States, there are approximately 2.9 million emergency department visits and 3.5 million outpatient visits annually due to urinary tract infections, with related expenditures estimated to exceed $6 billion and extensive use of antibiotics leading to an increase in antimicrobial resistance (2). Its risk factors include diabetes mellitus, chronic kidney disease, the use of immunosuppressants, renal transplantation, urinary tract catheterization, and neurogenic bladder (3). Urinary tract infections are caused by various pathogens, including Gram-positive bacteria, Gram-negative bacteria, and fungi. And in the case of bacterial infections, antibiotics are the core treatment (4). The choice of antibiotics and the duration of treatment vary depending on the different pathogens causing urinary tract infections (5). The abuse of antibiotics promotes drug resistance, making it particularly important to choose a more appropriate antibiotic as the initial treatment to rapidly alleviate symptoms and slow down disease progression (6). Since it often takes several days for the urine culture results of patients to be reported, some patients may require empirical treatment before these results are available (7). Besides, the empirical use of antibiotics often varies by region (8). However, in China, there is a paucity of relevant clinical data, and the data are changing rapidly. There is in need of studies to update the local status of urinary tract infections and antibiotic resistance patterns. In this study, we analyzed the causative organisms of urinary tract infections and their drug sensitivity in our center, which can help to guide the choice of initial antibiotics, reduce the occurrence of drug-resistant events, and improve the prognosis of patients locally.

## 2 Materials and methods

### 2.1 Patient data collection

Data of all patients with positive urine cultures with bacterial count more than 100,000 CFUs/mL from January 2022 to December 2023 in the Department of Urology, Wuhan Union Hospital were included in the study. The exclusion criteria were as follows: (1) a negative urine culture result; (2) a positive urine culture but the bacterial count is fewer than 10,000 CFUs/mL suggests contamination; (3) a positive urine culture but the bacterial count ranges from 10,000 to 100,000 CFUs/mL indicates further evaluation; (4) the absence of relevant data. A total of 3.685 positive urine culture data was included. The study was approved by the Ethics Committee of Wuhan Union Hospital.

### 2.2 Urine sample collection, microorganism identification, and antibiotic susceptibility testing

Urine sample collection required every patient collecting a clean-catch midstream urine sample in a sterile container. Once collected, samples were promptly sent for testing. Initial assessment guided the choice of pre-inoculation processing, culture conditions, and media such as blood agar plates, MacConkey agar, and Sabouraud dextrose agar. The identification utilized colony characteristics, bacterial morphology, staining properties, and biochemical reactions. In necessary cases, matrix-assisted laser desorption ionization time-of-flight mass spectrometry (MALDI-TOF MS) was applied for identification.

Antibiotic susceptibility testing was conducted employing the broth dilution method. After incubating, the minimum inhibitory concentration (MIC) could be identified. The results were interpreted according to Clinical and Laboratory Standards Institute (CLSI) standards, classifying them into sensitivity (S), resistance (R), intermediate (I), and in some cases, dose-dependent sensitivity (SDD).

### 2.3 Statistical methods

Categorical variables were expressed as rates or constitutive ratios and were compared using the *χ*^2^ test or Fisher’s exact test. Both the *χ*^2^ test and Fisher’s exact test were used to examine whether there were differences in the rates or constitutive ratios of categorical variables. Specifically, the *χ*^2^ test requires that the expected frequency in each cell is at least 5, whereas Fisher’s exact test is appropriate when the expected frequencies in some cells of the contingency table are less than 5. In all analyses, *p* < 0.05 was considered statistically significant.

## 3 Results

### 3.1 Basic patient profile

A total of 3,685 urine culture samples were positive, including 1,888 (51.23%) males and 1,797 (48.77%) females, and the mean age of patients was 56.09 years old. Besides, a total of 3,899 strains of pathogens were detected.

### 3.2 Classification and sensitivity rate of pathogens

**Figure 1:**
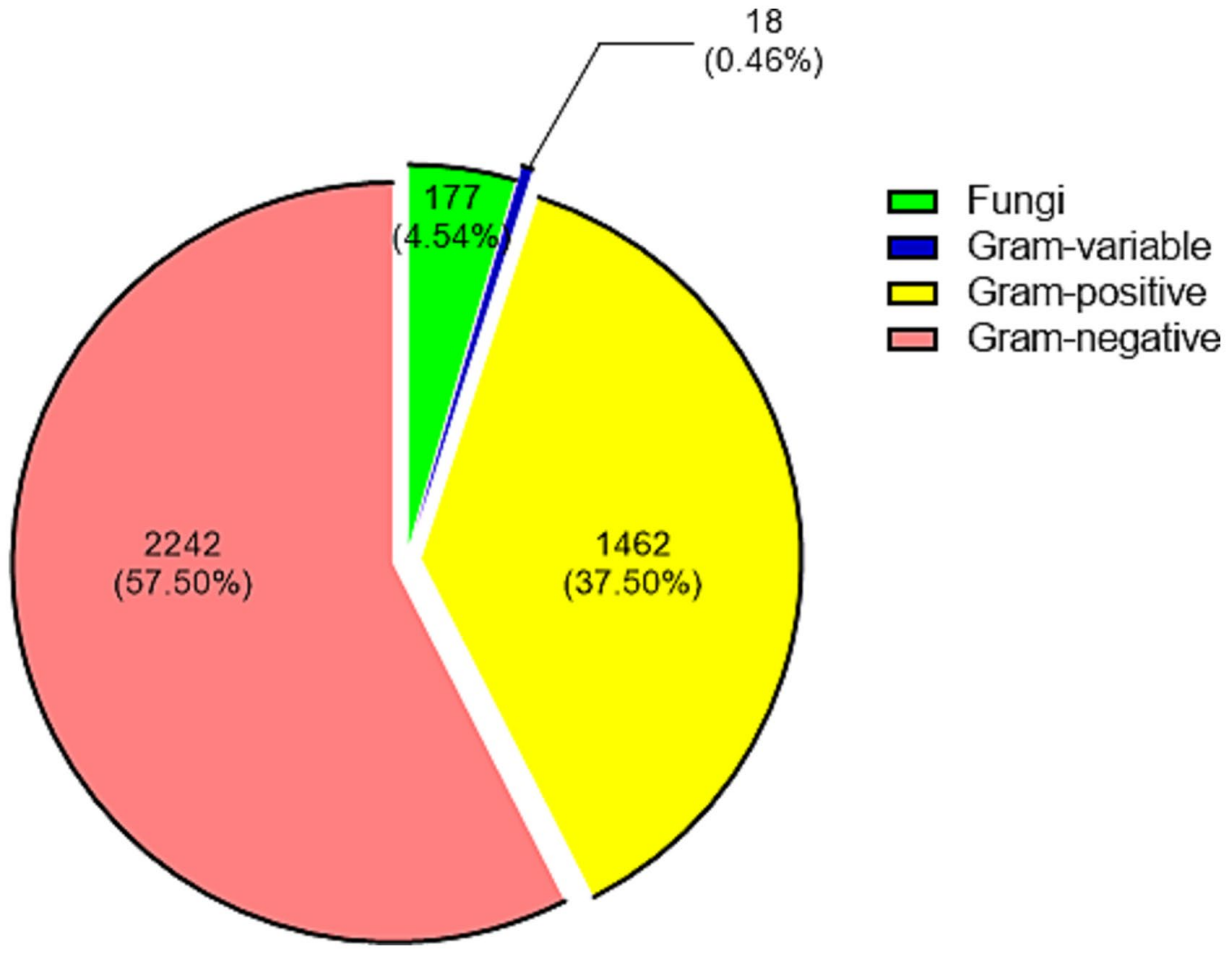
Distribution of 3,899 strains of causative organisms according to their classification. Each group is presented by count (proportion).

Out of 3,899 pathogens, 1,462 (37.50%) were Gram-positive, 2,242 (57.50%) were Gram-negative, 177 (4.54%) were fungi, and 18 (0.46%) were gram-variable (Figure 1). The top 10 Gram-positive bacteria, top 10 Gram-negative bacteria, and top 5 fungi were shown in Table 1. Comparing the Gram-positive and Gram-negative groups, the overall antibiotic susceptibility of the two groups was shown in Supplementary Table 1. For Gram-positive bacteria, Linezolid, Teicoplanin, and Vancomycin showed susceptibility rates of more than 97%, while for Gram-negative bacteria, Polymyxin B, Meropenem, Tigecycline, Ceftazidime/avibactam, and Colistin exhibit susceptibility rates exceeding 95%. The *χ*^2^ test for sensitivity to eight drugs were tested, with both Gram-positive and Gram-negative bacteria. It showed that the differences in susceptibility to ampicillin (*χ*^2^ = 878.8, *ν* = 1, *p* < 0.0001), cotrimoxazole (*χ*^2^ = 19.87, *ν* = 1, *p* < 0.0001), ciprofloxacin (*χ*^2^ = 30.51, *ν* = 1, *p* < 0.0001), minocycline (*χ*^2^ = 224.7, *ν* = 1, *p* < 0.0001), and ceftriaxone (*χ*^2^ = 82.75, ν = 1, *p* < 0.0001) were statistically significant, while others were not, including chloramphenicol (*χ*^2^ = 3.625, ν = 1, *p* = 0.0569), gentamicin (*χ*^2^ = 0.1631, ν = 1, *p* = 0.6863), and levofloxacin (*χ*^2^ = 1.279, ν = 1, *p* = 0.2581).

**Table 1:** Top 10 Gram-positive bacteria, top 10 Gram-negative bacteria, and top 5 fungi.

Rank	Gram-positive bacteria (*n* = 1,462)		Gram-negative bacteria (*n* = 2,242)		Fungi (*n* = 177)	
	Pathogen	Count (proportion)	Pathogen	Count (proportion)	Pathogen	Count (proportion)
1	*Enterococcus faecalis*	549 (37.55%)	*Escherichia coli*	1,250 (55.75%)	*Candida albicans*	75 (42.37%)
2	*Group B Streptococcus*	193 (13.20%)	*Klebsiella pneumoniae*	204 (9.10%)	*Candida parapsilosis*	36 (20.34%)
3	*Staphylococcus epidermidis*	156 (10.67%)	*Pseudomonas aeruginosa*	123 (5.49%)	*Candida tropicalis*	27 (15.25%)
4	*Enterococcus faecium*	136 (9.30%)	*Proteus mirabilis*	122 (5.44%)	*Candida glabrata*	22 (12.43%)
5	*Viridans group streptococci*	100 (6.84%)	*Acinetobacter baumannii*	80 (3.57%)	*Candida krusei*	6 (3.39%)
6	*Hemolytic staphylococci*	67 (4.58%)	*Enterobacter cloacae*	76 (3.39%)		
7	*Corynebacterium* spp.	42 (2.87%)	*Morganella morganii*	48 (2.14%)		
8	*Staphylococcus aureus*	41 (2.80%)	*Klebsiella oxytoca*	37 (1.65%)		
9	*Corynebacterium glucuronolyticum*	21 (1.44%)	*Enterobacter aerogenes*	33 (1.47%)		
10	*Streptococcus oralis*	19 (1.30%)	*Citrobacter freundii*	29 (1.29%)		

The *n* means the total count in each group.

### 3.3 Classification and sensitivity rate of urine culture-positive stones

**Figure 2:**
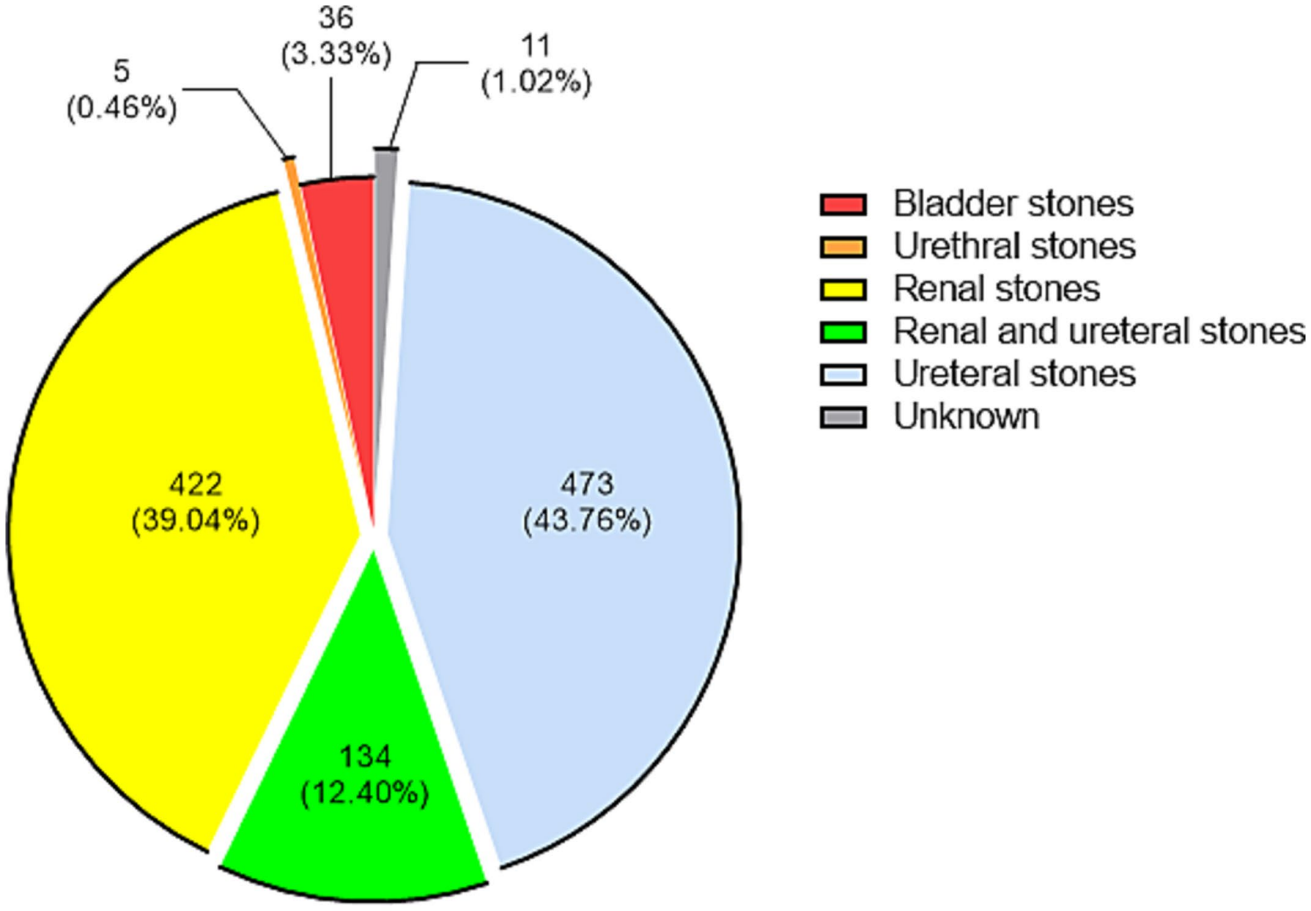
Distribution of 1,081 stone-associated pathogens according to their stone position. Each group is presented by count (proportion).

Of the 3,899 pathogens, 1,081 (27.73%) were stone-associated and 2,818 (72.27%) were non-stone-associated, and the top 10 pathogens in the two groups were shown in Table 2. The *χ*^2^ test for the composition of the top 10 pathogens in the two groups showed that the difference was statistically significant only for *Proteus mirabilis* (*χ*^2^ = 15.53, *ν* = 1, *p* < 0.0001). The positions of stones were categorized according to the diagnosis, with the results of 473 ureteral stones (43.76%), 422 renal stones (39.04%), 134 renal and ureteral stones (12.40%), 36 bladder stones (3.33%), and 5 urethral stones (0.46%), and the positions of the remaining 11 were not known (Figure 2). The top 10 pathogens for ureteral stones, renal and ureteral stones, and renal stones were shown in Supplementary Table 2. In these three positions of stones, *Escherichia coli* is the most common pathogen, with proportions of 31.50, 27.61, and 36.97%, respectively.

**Table 2:** Top 10 pathogens in different groups from three aspects.

Rank	Stone-associated (*n* = 1,081)		Non-stone-associated (*n* = 2,818)		2022 (*n* = 1704)		2023 (*n* = 2,195)		Outpatient clinic (*n* = 523)		Ward (*n* = 3,376)	
	Pathogen	Count (Proportion)	Pathogen	Count (Proportion)	Pathogen	Count (Proportion)	Pathogen	Count (Proportion)	Pathogen	Count (Proportion)	Pathogen	Count (Proportion)
1	*Escherichia coli*	351 (32.47%)	*Escherichia coli*	899 (31.90%)	***Escherichia coli***	**507 (29.75%)**	***Escherichia coli***	**743 (33.85%)**	***Escherichia coli***	**244 (46.65%)**	***Escherichia coli***	**1,006 (29.80%)**
2	*Enterococcus faecalis*	139 (12.86%)	*Enterococcus faecalis*	410 (14.55%)	*Enterococcus faecalis*	243 (14.26%)	*Enterococcus faecalis*	306 (13.94%)	***Enterococcus faecalis***	**55 (10.52%)**	***Enterococcus faecalis***	**494 (14.63%)**
3	*Group B Streptococcus*	65 (6.01%)	*Klebsiella pneumoniae*	151 (5.36%)	*Klebsiella pneumoniae*	94 (5.52%)	*Klebsiella pneumoniae*	110 (5.01%)	***Klebsiella pneumoniae***	**39 (7.46%)**	***Klebsiella pneumoniae***	**165 (4.89%)**
4	*Klebsiella pneumoniae*	53 (4.90%)	*Group B Streptococcus*	128 (4.54%)	*Group B Streptococcus*	84 (4.93%)	*Group B Streptococcus*	109 (4.97%)	*Group B Streptococcus*	30 (5.74%)	*Group B Streptococcus*	163 (4.83%)
5	***Proteus mirabilis***	**53 (4.90%)**	*Staphylococcus epidermidis*	118 (4.19%)	***Enterococcus faecium***	**71 (4.17%)**	*Staphylococcus epidermidis*	90 (4.10%)	*Viridans group streptococci*	12 (2.29%)	***Staphylococcus epidermidis***	**146 (4.32%)**
6	*Enterococcus faecium*	44 (4.07%)	*Enterococcus faecium*	92 (3.26%)	*Staphylococcus epidermidis*	66 (3.87%)	***Proteus mirabilis***	**88 (4.01%)**	*Proteus mirabilis*	11 (2.10%)	***Enterococcus faecium***	**127 (3.76%)**
7	*Staphylococcus epidermidis*	38 (3.52%)	*Pseudomonas aeruginosa*	91 (3.23%)	*Pseudomonas aeruginosa*	58 (3.40%)	*Pseudomonas aeruginosa*	65 (2.96%)	***Staphylococcus epidermidis***	**10 (1.91%)**	***Pseudomonas aeruginosa***	**115 (3.41%)**
8	*Viridans group streptococci*	32 (2.96%)	***Proteus mirabilis***	**69 (2.45%)**	*Viridans group streptococci*	46 (2.70%)	***Enterococcus faecium***	**65 (2.96%)**	*Candida albicans*	9 (1.72%)	*Proteus mirabilis*	111 (3.29%)
9	*Pseudomonas aeruginosa*	32 (2.96%)	*Viridans group streptococci*	68 (2.41%)	*Enterobacter cloacae*	40 (2.35%)	*Viridans group streptococci*	54 (2.46%)	***Enterobacter aerogenes***	**9 (1.72%)**	*Viridans group streptococci*	88 (2.61%)
10	*Acinetobacter baumannii*	17 (1.57%)	*Acinetobacter baumannii*	63 (2.24%)	*Acinetobacter baumannii*	38 (2.23%)	*Hemolytic staphylococci*	42 (1.91%)	***Enterococcus faecium***	**9 (1.72%)**	***Acinetobacter baumannii***	**79 (2.34%)**

The *n* means the total count in each group. Bold text indicates statistical significance.

### 3.4 Association between urine culture-positive stones and the incidence of systemic inflammatory response syndrome (SIRS)

A total of 701 patients with urine culture-positive stones underwent lithotripsy in the Department of Urology of our hospital, and a total of 740 strains of causative organisms were isolated by urine culture. Thirty-seven patients had a dual infection and 1 patient had a triple infection. Seventy-five of these patients were diagnosed with SIRS after surgery, and a total of 79 strains of pathogens were isolated from the urine cultures of these patients. The occurrence rate of postoperative SIRS due to different types of pathogen infections was shown in Supplementary Table 3, and the difference in their proportion was statistically significant (*χ*^2^ = 13.87, *ν* = 2, *p* = 0.001). The ranking of pathogens (≥2 strains) isolated by urine culture from patients who suffered from SIRS after lithotripsy was shown in Supplementary Table 4.

### 3.5 Pathogens of urinary tract infections between 2022 and 2023

A total of 1,704 (43.70%) pathogens were isolated by urine culture in 2022, whereas a total of 2,195 (56.30%) pathogens were isolated by urine culture in 2023, and the top 10 pathogens in the two groups were shown in Table 2. Among them, there was a statistically significant difference between *E. coli* (*χ*^2^ = 7.389, *ν* = 1, *p* = 0.0066), *Enterococcus faecium* (*χ*^2^ = 4.140, ν = 1, *p* = 0.0419), and *P. mirabilis* (*χ*^2^ = 12.83, ν = 1, *p* = 0.0003).

### 3.6 Causative organisms of urinary tract infection between the outpatient clinic and ward

A total of 523 (13.41%) pathogens were isolated from urine cultures of the outpatient clinic, whereas 3,376 (86.59%) pathogens were isolated from urine cultures of the ward. The top 10 pathogens in both groups were shown in Table 2. *χ*^2^ test was performed on the proportion of the pathogens appearing in the table, in which the difference between *E. coli* (*χ*^2^ = 59.07, *ν* = 1, *p* < 0.0001), *Enterococcus faecalis* (*χ*^2^ = 6.343, ν = 1, *p* = 0.0118), *Klebsiella pneumoniae* (*χ*^2^ = 6.030, ν = 1, *p* = 0.0141), *Staphylococcus epidermidis* (*χ*^2^ = 6.863, ν = 1, *p* = 0.0088), *Enterobacter aerogenes* (*χ*^2^ = 5.504, ν = 1, *p* = 0.0190), *E. faecium* (*χ*^2^ = 5.604, ν = 1, *p* = 0.0179), *Pseudomonas aeruginosa* (*χ*^2^ = 5.221, ν = 1, *p* = 0.0223), and *Acinetobacter baumannii* (*p* = 0.0002) was found to be statistically significant.

### 3.7 Drug susceptibility rate of common pathogens of urinary tract infection

The four common bacteria of urinary tract infection, including *E. coli*, *K. pneumoniae*, *E. faecalis*, and *group B streptococcus*, were again divided into different subgroups: (1) Stone-associated and non-stone-associated; (2) Year of 2022 and 2023; (3) Outpatient clinic and ward. For each bacterium, antimicrobial susceptibility was described (Supplementary Tables 5–8). For *E. coli*, drugs like Amikacin, Meropenem, Tigecycline, Ceftazidime/avibactam, and Imipenem exhibited high susceptibility rates for nearly 100%. *K. pneumoniae* exhibited over 95% susceptibility rates against Tigecycline, Ceftazidime/avibactam, and Colistin. *E. faecalis* showed very high susceptibility to Ampicillin, Nitrofurantoin, Linezolid, Penicillin G, Teicoplanin, and Vancomycin. Lastly, Group B Streptococcus maintains 100% susceptibility to Linezolid, Penicillin G, Ceftriaxone, and Vancomycin.

## 4 Discussion

The data showed that Gram-negative bacteria were more common in urinary tract infections, and most of them were usually found in the digestive system. The common Gram-positive bacteria included *E. faecalis* and *group B Streptococcus*, and the common Gram-negative bacteria included *Escherichia coli* and *K. pneumoniae*, which was almost the same as the proportion of pathogens of urinary tract infections proved by other studies (9). Among Gram-negative bacteria, high susceptibility was observed to amikacin, piperacillin/tazobactam, and cefoperazone/sulbactam, supporting their potential use in empirical therapy. Among them, cefoperazone/sulbactam, piperacillin/tazobactam, and amikacin are recommended for *E. coli*. Alternatives can be meropenem, tigecycline, ceftazidime avibactam, and imipenem. For *K. pneumoniae*, amikacin is preferred, followed by cefoperazone/sulbactam and piperacillin/tazobactam. Meropenem, imipenem, tigecycline, and ceftazidime/avibactam can be alternatives. For Gram-positive bacteria, their composition was complex and their drug resistance varied greatly. Their drug sensitivity tests only chose a few antibiotics, so only ampicillin, nitrofurantoin, and ceftriaxone are recommended, and linezolid, vancomycin, and teicoplanin can be used as alternatives. For *E. faecalis*, ampicillin, nitrofurantoin, and penicillin G are recommended, while linezolid, teicoplanin, and vancomycin can be used as alternatives. For *group B streptococci*, ceftriaxone and penicillin G are recommended, while linezolid and vancomycin can be used as alternatives. In all, cephalosporins were found to be particularly effective against urinary tract infections, especially ceftriaxone, which demonstrated broad coverage for both Gram-negative and some Gram-positive bacteria. This makes cephalosporins a preferred choice for initial empirical treatment.

Data from Saudi Arabia indicated that non-diabetic urinary tract infection patients exhibited the highest resistance to amoxicillin and ampicillin, with approximately 40% resistance rates. In contrast, diabetic urinary tract infection patients showed the strongest resistance to tetracycline and cephalosporins, both exceeding 40% (10). In addition, a study in Ethiopia showed that the majority of bacteria isolated from patients with urinary tract stones had resistance rates exceeding 90% to ampicillin, 60% to penicillin, and 40% to trimethoprim-sulfamethoxazole (11). Another interesting study found that the resistance rates to nitrofurantoin, cefazolin, ciprofloxacin, and trimethoprim-sulfamethoxazole in women aged ≥18 years with urinary tract infections were all between 10 and 20%. Additionally, women with a history of kidney stones may exhibit bacterial urine resistance to nitrofurantoin (12). Some of the data mentioned above differ significantly from the data collected in our center. In comparison, our center has observed higher resistance rates for certain types of antibiotics, underscoring the regional variations in antibiotic resistance. Empirical treatment practices often vary by region, and there is a need for more meaningful statistics to guide such practices locally.

By analyzing the urine culture results of patients with lithiasis, it could be found that the top 10 bacterial types were the same, but their proportions and sequences have changed. *E. coli* and *E. faecalis* were the most common pathogens in both stone and non-stone-associated urinary tract infections. Interestingly, the magnesium ammonium phosphate stones, also known as infected stones, were mainly caused by urea-splitting bacteria such as *P. mirabilis*, which may explain the statistically significant difference of *P. mirabilis* between stone and non-stone urinary tract infections, so we must put greater demand on the targeted administration of antibiotics for patients with infected stones (13–15). Different stone locations appear to harbor distinct bacterial populations, suggesting site-specific preferences. For patients undergoing lithotripsy with positive urine cultures, the risk of suffering from SIRS postoperatively appeared to be related to the types of pathogens. Although fungal infections, particularly *Candida glabrata*, were associated with a numerically higher rate of postoperative SIRS, the small number of cases limits the strength of this observation, and only one study reported similar findings (16). Nonetheless, given the potential severity, early detection and timely therapy are essential to prevent SIRS.

The comparison of the proportion of pathogens in urine culture in 2022 and 2023 showed that *E. coli* was the most common pathogen in both years. The observed shifts in pathogen distribution between 2022 and 2023 highlight the dynamic nature of urinary tract infection epidemiology. While external factors such as the COVID-19 pandemic may have contributed. This underscores the need for dynamic adjustment of empirical treatment protocols based on current epidemiological data (17). When comparing the distribution of pathogens between outpatient clinics and wards, significant differences were observed. On one hand, the result may be due to the smaller sample size in the outpatient clinics. On the other hand, it may be due to differences in the disease spectrum between outpatient and inpatient settings, indicating that urological diseases may have a certain predisposition toward certain pathogens. This would require further in-depth research for validation, and differences in bacterial spectrum should also be taken into account in the empirical selection of antibiotics in hospitalized patients. In addition, most hospitalized patients have indications for surgery, and the correct use of antimicrobial drugs and obtaining urine culture results as soon as possible are of great significance in preventing severe infections after surgery.

This study had several limitations, including potential data bias due to patient’s non-compliance with proper procedures, small sample size in certain subgroups, and data from a single center. These factors highlight the need for further multicenter prospective studies to validate our findings and provide more generalizable guidelines for clinical practice. Besides, our study primarily relied on χ2 tests, and future studies incorporating multivariable modeling are needed to adjust for potential confounders.

In conclusion, based on the above results, it is recommended to prioritize cephalosporin antibiotics in the initial empirical treatment and use sensitive antimicrobial drugs in a timely manner according to the results of antimicrobial susceptibility testing. This could avoid the indiscriminate use of broad-spectrum antibiotics to prevent the escalation of resistance. Further multicenter prospective studies and mechanism experiments are needed in the future to observe longitudinal trends in antibiotic efficacy and guide clinical practice.

## Data Availability

The raw data supporting the conclusions of this article will be made available by the authors, without undue reservation.

## References

[1] Vallo S Wirth P Kukic A Nafez O Neagoe L Nestler S. Decreasing susceptibility of bacteria to ampicillin/ sulbactam and third generation cephalosporins in urinary tract infections. Curr Urol. (2022) 16:94–8. doi: 10.1097/CU9.0000000000000079, PMID: 36601280 PMC9782476

[2] Timm MR Russell SK Hultgren SJ. Urinary tract infections: pathogenesis, host susceptibility and emerging therapeutics. Nat Rev Microbiol. (2025) 23:72–86. doi: 10.1038/s41579-024-01092-4, PMID: 39251839 PMC13194463

[3] Alghoraibi H Asidan A Aljawaied R Almukhayzim R Alsaydan A Alamer E. Recurrent urinary tract infection in adult patients, risk factors, and efficacy of low dose prophylactic antibiotics therapy. J Epidemiol Glob Health. (2023) 13:200–11. doi: 10.1007/s44197-023-00105-4, PMID: 37273158 PMC10271986

[4] Zhou Y Zhou Z Zheng L Gong Z Li Y Jin Y. Urinary tract infections caused by Uropathogenic *Escherichia coli*: mechanisms of infection and treatment options. Int J Mol Sci. (2023) 24. doi: 10.3390/ijms241310537 PMC10341809 37445714

[5] Al Lawati H Blair BM Larnard J. Urinary tract infections: Core curriculum 2024. Am J Kidney Dis. (2024) 83:90–100. doi: 10.1053/j.ajkd.2023.08.009, PMID: 37906240

[6] Brodie A El-Taji O Jour I Foley C Hanbury D. A retrospective study of immunotherapy treatment with Uro-Vaxom (OM-89®) for prophylaxis of recurrent urinary tract infections. Curr Urol. (2020) 14:130–4. doi: 10.1159/000499248, PMID: 33224005 PMC7659410

[7] Kurotschka PK Gágyor I Ebell MH. Acute uncomplicated UTIs in adults: rapid evidence review. Am Fam Physician. (2024) 109:167–74. 38393801

[8] Moja L Zanichelli V Mertz D Gandra S Cappello B Cooke GS. WHO’S essential medicines and AWaRe: recommendations on first- and second-choice antibiotics for empiric treatment of clinical infections. Clin Microbiol Infect. (2024) 30:S1–s51. doi: 10.1016/j.cmi.2024.02.003 38342438

[9] Alhhazmi AA Alhazmi RA Alahmadi ES Almuallim WM Aljurfi ZI Alturkostani MA. Prevalence and resistance patterns of urinary tract infection in Al-Madinah Al-Munawarah, Saudi Arabia: a retrospective study. Discov Med. (2024) 36:853–64. doi: 10.24976/Discov.Med.202436183.80 38665033

[10] Farag PF Albulushi HO Eskembaji MH Habash MF Malki MS Albadrani MS. Prevalence and antibiotic resistance profile of UTI-causing uropathogenic bacteria in diabetics and non-diabetics at the maternity and children Hospital in Jeddah, Saudi Arabia. Front Microbiol. (2024) 15:1507505. doi: 10.3389/fmicb.2024.1507505 39669784 PMC11635965

[11] Kasew D Eshetie S Diress A Tegegne Z Moges F. Multiple drug resistance bacterial isolates and associated factors among urinary stone patients at the University of Gondar Comprehensive Specialized Hospital, Northwest Ethiopia. BMC Urol. (2021) 21:27. doi: 10.1186/s12894-021-00794-8, PMID: 33622301 PMC7901194

[12] Mohseni M Craver EC Heckman MG Sheele JM. Can urinalysis and past medical history of kidney stones predict urine antibiotic resistance? West J Emerg Med. (2022) 23:613–7. doi: 10.5811/westjem.2022.4.54872, PMID: 36205684 PMC9541996

[13] Das P Gupta G Velu V Awasthi R Dua K Malipeddi H. Formation of struvite urinary stones and approaches towards the inhibition-a review. Biomed Pharmacother. (2017) 96:361–70. doi: 10.1016/j.biopha.2017.10.015, PMID: 29028588

[14] Norsworthy AN Pearson MM. From catheter to kidney stone: the Uropathogenic lifestyle of *Proteus mirabilis*. Trends Microbiol. (2017) 25:304–15. doi: 10.1016/j.tim.2016.11.015, PMID: 28017513 PMC5365347

[15] Smith SN Armbruster CE. Indwelling urinary catheter model of *Proteus mirabilis* infection. Methods Mol Biol. (2019) 2021:187–200. doi: 10.1007/978-1-4939-9601-8_17, PMID: 31309506

[16] Yecies T Mohapatra A Semins MJ. Outcomes of Endourologic interventions in patients with preoperative Funguria. J Endourol. (2019) 33:668–72. doi: 10.1089/end.2019.0371 30924689

[17] Kuitunen I Artama M Haapanen M Renko M. Urinary tract infections decreased in Finnish children during the COVID-19 pandemic. Eur J Pediatr. (2022) 181:1979–84. doi: 10.1007/s00431-022-04389-9, PMID: 35098402 PMC8801286

