# Supplementary material for: Causative organisms of urinary tract infections and their drug sensitivity: an analysis from various aspects
Source: Front Public Health. 2025 Oct 29;13:1487721. doi: 10.3389/fpubh.2025.1487721 (PMC12605273; doi:10.3389/fpubh.2025.1487721)
Supplement: Supplementary file 1 [file Table_1.DOCX]

**Supplementary table 1. Overall antibiotic susceptibility of gram-positive bacteria and gram-negative bacteria**

| Gram-positive bacteria | | Gram-negative bacteria | |
| --- | --- | --- | --- |
| Antibiotics | Susceptibility | Antibiotics | Susceptibility |
| **Ampicillin** | **80.03%（549/686）** | Amikacin | 94.86%（1995/2103） |
| Oxacillin | 44.52%（138/310） | Amoxicillin/clavulanate | 68.21%（1118/1639） |
| Nitrofurantoin | 80.65%（550/682） | **Ampicillin** | **13.58%（186/1370）** |
| **Cotrimoxazole** | **68.79%（216/314）** | Ampicillin/sulbactam | 51.85%（885/1707） |
| Gentamicin (high level) | 81.18%（535/659） | Aztreonam | 59.68%（1202/2014） |
| Erythromycin | 15.57%（208/1336） | Polymyxin B | 98.82%（84/85） |
| Ciprofloxacin | 31.90%（318/997） | **Cotrimoxazole** | **55.40%（1103/1991）** |
| Clindamycin | 40.63%（260/640） | Ciprofloxacin | 42.27%（880/2082） |
| Linezolid | 100.00%（1315/1315） | Chloramphenicol | 64.76%（1180/1822） |
| Chloramphenicol | 68.81%（470/683） | Meropenem | 95.51%（2001/2095） |
| **Minocycline** | **45.92%（456/993）** | **Minocycline** | **74.50%（1300/1745）** |
| Penicillin G | 61.07%（811/1328） | Piperacillin | 31.25%（631/2019） |
| Gentamicin | 64.97%（204/314） | Piperacillin/tazobactam | 86.92%（1827/2102） |
| Teicoplanin | 100.00%（683/683） | Gentamicin | 66.13%（1386/2096） |
| **Ceftriaxone** | **92.65%（315/340）** | Tetracycline | 48.80%（832/1705） |
| Vancomycin | 97.75%（1301/1331） | Tigecycline | 98.93%（1659/1677） |
| Levofloxacin | 51.65%（532/1030） | Cefepime | 57.20%（1203/2103） |
|  |  | Cefuroxime | 41.54%（27/65） |
|  |  | Cefoperazone/sulbactam | 88.51%（1648/1862） |
|  |  | **Ceftriaxone** | **52.94%（45/85）** |
|  |  | Cefotaxime | 45.79%（865/1889） |
|  |  | Ceftazidime | 70.75%（1500/2120） |
|  |  | Ceftazidime/avibactam | 99.52%（1875/1884） |
|  |  | Cefoxitin | 75.00%（51/68） |
|  |  | Cefazolin | 20.61%（249/1208） |
|  |  | Tobramycin | 54.22%（45/83） |
|  |  | Imipenem | 89.15%（1833/2056） |
|  |  | Colistin | 95.20%（1724/1811） |
|  |  | Levofloxacin | 49.50%（1037/2095） |

Bold text indicates statistical significance.

**Supplementary table 2. Top 10 pathogens for ureteral stones, renal and ureteral stones, and renal stones**

|  | Ureteral stones（n=473） | | Renal and ureteral stones（n=134） | | Renal stones（n=422） | |
| --- | --- | --- | --- | --- | --- | --- |
|  | Pathogen | Count (Proportion) | Pathogen | Count (Proportion) | Pathogen | Count (Proportion) |
| 1 | *Escherichia coli* | 149 (31.50%) | *Escherichia coli* | 37 (27.61%) | *Escherichia coli* | 156 (36.97%) |
| 2 | *Enterococcus faecalis* | 73 (15.43%) | *Enterococcus faecalis* | 18 (13.43%) | *Enterococcus faecalis* | 39 (9.24%) |
| 3 | *Group B Streptococcus* | 32 (6.77%) | *Group B Streptococcus* | 13 (9.70%) | *Proteus mirabilis* | 31 (7.35%) |
| 4 | *Staphylococcus epidermidis* | 21 (4.44%) | *Enterococcus faecium* | 7 (5.22%) | *Klebsiella pneumoniae* | 22 (5.21%) |
| 5 | *Klebsiella pneumoniae* | 19 (4.02%) | *Klebsiella pneumoniae* | 7 (5.22%) | *Enterococcus faecium* | 22 (5.21%) |
| 6 | *Viridans group streptococci* | 17 (3.59%) | *Pseudomonas aeruginosa* | 7 (5.22%) | *Group B Streptococcus* | 20 (4.74%) |
| 7 | *Proteus mirabilis* | 15 (3.17%) | *Viridans group streptococci* | 4 (2.99%) | *Pseudomonas aeruginosa* | 15 (3.55%) |
| 8 | *Enterococcus faecium* | 13 (2.75%) | *Enterobacter cloacae* | 4 (2.99%) | *Staphylococcus epidermidis* | 12 (2.84%) |
| 9 | *Hemolytic staphylococci* | 9 (1.90%) | *Proteus mirabilis* | 4 (2.99%) | *Viridans group streptococci* | 10 (2.37%) |
| 10 | *Pseudomonas aeruginosa* | 8 (1.69%) | *Corynebacterium glucuronolyticum* | 3 (2.24%) | *Acinetobacter baumannii* | 7 (1.66%) |

The n means the total count in each group.

**Supplementary table 3. Postoperative SIRS due to different types of pathogen infections**

|  | SIRS | Total | Occurrence rate |
| --- | --- | --- | --- |
| Gram-positive bacteria | 16 | 283 | 5.60% |
| Gram-negative bacteria | 57 | 424 | 13.40% |
| Fungi | 6 | 29 | 20.70% |

**Supplementary table 4. Pathogens (≥2 strains) isolated by urine culture from patients suffered from SIRS after lithotripsy**

|  | Fungi | | | | Gram-positive bacteria | | | | Gram-negative bacteria | | | |
| --- | --- | --- | --- | --- | --- | --- | --- | --- | --- | --- | --- | --- |
|  | Pathogen | SIRS | Total | Occurrence rate | Pathogen | SIRS | Total | Occurrence rate | Pathogen | SIRS | Total | Occurrence rate |
| 1 | *Candida albicans* | 2 | 8 | 25.00% | *Enterococcus faecalis* | 9 | 92 | 9.78% | *Escherichia coli* | 39 | 261 | 14.94% |
| 2 | *Candida glabrata* | 3 | 6 | 50.00% | *Enterococcus faecium* | 3 | 30 | 10.00% | *Proteus mirabilis* | 4 | 34 | 11.76% |
| 3 |  |  |  |  |  |  |  |  | *Klebsiella pneumoniae* | 3 | 30 | 10.00% |
| 4 |  |  |  |  |  |  |  |  | *Pseudomonas aeruginosa* | 4 | 16 | 25.00% |
| 5 |  |  |  |  |  |  |  |  | *Acinetobacter baumannii* | 2 | 11 | 18.18% |
| 6 |  |  |  |  |  |  |  |  | *Citrobacter freundii* | 2 | 10 | 20.00% |

**Supplementary table 5. Results of Escherichia coli antimicrobial susceptibility testing.**

| Antibiotics | Stone-associated | Non-stone-associated | 2022 | 2023 | Outpatient clinic | Ward | Total |
| --- | --- | --- | --- | --- | --- | --- | --- |
| Amikacin | 95.49% (339/355) | 96.45% (788/817) | 96.47% (464/481) | 95.95% (663/691) | 95.92% (965/1006) | 97.59% (162/166) | 96.16% (1127/1172) |
| Amoxicillin/clavulanate | 64.79% (230/355) | 68.46% (560/818) | 63.69% (307/482) | 69.90% (483/691) | 66.60% (670/1006) | 71.86% (120/167) | 67.35% (790/1173) |
| Ampicillin | 6.52% (23/353) | 12.47% (102/818) | 10.58% (51/482) | 10.74% (74/689) | 10.46% (105/1004) | 11.98% (20/167) | 10.67% (125/1171) |
| Ampicillin/sulbactam | 43.20% (146/338) | 47.08% (371/788) | 28.17% (131/465) | 58.40% (386/661) | 45.13% (436/966) | 50.63% (81/160) | 45.91% (517/1126) |
| Aztreonam | 47.04% (167/355) | 56.43% (461/817) | 53.85% (259/481) | 53.40% (369/691) | 51.99% (523/1006) | 63.25% (105/166) | 53.58% (628/1172) |
| Polymyxin B | 100.00% (14/14) | 100.00% (26/26) | 100.00% (17/17) | 100.00% (23/23) | 100.00% (34/34) | 100.00% (6/6) | 100.00% (40/40) |
| Cotrimoxazole | 44.63% (158/354) | 49.39% (403/816) | 45.21% (217/480) | 49.86% (344/690) | 48.80% (490/1004) | 42.77% (71/166) | 47.95% (561/1170) |
| Ciprofloxacin | 23.14% (81/350) | 29.61% (241/814) | 26.78% (128/478) | 28.28% (194/686) | 27.35% (273/998) | 29.52% (49/166) | 27.66% (322/1164) |
| Chloramphenicol | 73.00% (246/337) | 67.51% (532/788) | 64.30% (299/465) | 72.58% (479/660) | 68.60% (662/965) | 72.50% (116/160) | 69.16% (778/1125) |
| Meropenem | 98.86% (348/352) | 97.79% (795/813) | 98.33% (470/478) | 97.96% (673/687) | 98.30% (982/999) | 96.99% (161/166) | 98.11% (1143/1165) |
| Minocycline | 78.29% (274/350) | 75.21% (613/815) | 72.50% (348/480) | 78.69% (539/685) | 76.18% (761/999) | 75.90% (126/166) | 76.14% (887/1165) |
| Piperacillin | 7.04% (25/355) | 13.69% (112/818) | 11.62% (56/482) | 11.72% (81/691) | 11.33% (114/1006) | 13.77% (23/167) | 11.68% (137/1173) |
| Piperacillin/tazobactam | 89.30% (317/355) | 92.18% (754/818) | 92.53% (446/482) | 90.45% (625/691) | 90.85% (914/1006) | 94.01% (157/167) | 91.30% (1071/1173) |
| Gentamicin | 58.87% (209/355) | 60.47% (494/817) | 57.68% (278/482) | 61.59% (425/690) | 60.30% (606/1005) | 58.08% (97/167) | 59.98% (703/1172) |
| Tetracycline | 43.79% (148/338) | 42.26% (333/788) | 39.78% (185/465) | 44.78% (296/661) | 42.96% (415/966) | 41.25% (66/160) | 42.72% (481/1126) |
| Tigecycline | 99.72% (351/352) | 100.00% (809/809) | 100.00% (477/477) | 99.85% (683/684) | 99.90% (995/996) | 100.00% (165/165) | 99.91% (1160/1161) |
| Cefepime | 40.00% (142/355) | 47.68% (390/818) | 43.98% (212/482) | 46.31% (320/691) | 44.43% (447/1006) | 50.90% (85/167) | 45.35% (532/1173) |
| Cefuroxime | 31.25% (5/16) | 54.55% (18/33) | 58.82% (10/17) | 40.63% (13/32) | 47.50% (19/40) | 44.44% (4/9) | 46.94% (23/49) |
| Cefoperazone/sulbactam | 92.66% (328/354) | 90.45% (739/817) | 88.54% (425/480) | 92.91% (642/691) | 90.74% (911/1004) | 93.41% (156/167) | 91.12% (1067/1171) |
| Ceftriaxone | 58.82% (10/17) | 60.00% (18/30) | 64.71% (11/17) | 56.67% (17/30) | 57.50% (23/40) | 71.43% (5/7) | 59.57% (28/47) |
| Cefotaxime | 33.14% (112/338) | 43.65% (344/817) | 38.92% (181/465) | 41.60% (275/661) | 39.75% (384/966) | 45.00% (72/160) | 40.50% (456/1126) |
| Ceftazidime | 63.66% (226/355) | 67.36% (551/818) | 63.90% (308/482) | 67.87% (469/691) | 65.41% (658/1006) | 71.26% (119/167) | 66.24% (777/1173) |
| Ceftazidime/avibactam | 100.00% (351/351) | 99.50% (794/798) | 99.58% (471/473) | 99.70% (674/676) | 99.70% (982/985) | 99.39% (163/164) | 99.65% (1145/1149) |
| Cefoxitin | 70.59% (12/17) | 100.00% (30/30) | 94.12% (16/17) | 86.67% (26/30) | 87.50% (35/40) | 100.00% (7/7) | 89.36% (42/47) |
| Cefazolin | 15.81% (46/291) | 19.80% (121/611) | 1.88% (6/320) | 27.66% (161/582) | 17.73% (139/784) | 23.73% (28/118) | 18.51% (167/902) |
| Tobramycin | 41.18% (7/17) | 53.33% (16/30) | 64.71% (11/17) | 40.00% (12/30) | 45.00% (18/40) | 71.43% (5/7) | 48.94% (23/47) |
| Imipenem | 98.87% (351/355) | 97.07% (794/818) | 96.68% (466/482) | 98.26% (679/691) | 97.61% (982/1006) | 97.60% (163/167) | 97.61% (1145/1173) |
| Colistin | 98.22% (331/337) | 98.72% (774/784) | 98.49% (457/464) | 98.63% (648/657) | 98.44% (948/963) | 99.37% (157/158) | 98.57% (1105/1121) |
| Levofloxacin | 32.66% (114/349) | 36.90% (300/813) | 36.06% (172/477) | 35.33% (242/685) | 35.34% (352/996) | 37.35% (62/166) | 35.63% (414/1162) |

**Supplementary table 6. Results of Klebsiella pneumoniae antimicrobial susceptibility testing.**

| Antibiotics | Stone-associated | Non-stone-associated | 2022 | 2023 | Outpatient clinic | Ward | Total |
| --- | --- | --- | --- | --- | --- | --- | --- |
| Amikacin | 88.24% (45/51) | 89.58% (129/144) | 87.64% (78/89) | 90.57% (96/106) | 89.09% (147/165) | 90.00% (27/30) | 89.23% (174/195) |
| Amoxicillin/clavulanate | 72.55% (37/51) | 58.33% (84/144) | 55.06% (49/89) | 67.92% (72/106) | 61.21% (101/165) | 66.67% (20/30) | 62.05% (121/195) |
| Ampicillin/sulbactam | 63.83% (30/47) | 48.92% (68/139) | 46.43% (39/84) | 57.84% (59/102) | 53.85% (84/156) | 46.67% (14/30) | 52.69% (98/186) |
| Aztreonam | 62.75% (32/51) | 59.72% (86/144) | 56.18% (50/89) | 64.15% (68/106) | 59.39% (98/165) | 66.67% (20/30) | 60.51% (118/195) |
| Ampicillin/sulbactam | 66.67% (34/51) | 54.86% (79/144) | 55.06% (49/89) | 60.38% (64/106) | 58.18% (96/165) | 56.67% (17/30) | 57.95% (113/195) |
| Aztreonam | 53.06% (26/49) | 38.30% (54/141) | 36.78% (32/87) | 46.60% (48/103) | 41.98% (68/162) | 42.86% (12/28) | 42.11% (80/190) |
| Ampicillin/sulbactam | 61.70% (29/47) | 60.43% (84/139) | 55.95% (47/84) | 64.71% (66/102) | 61.54% (96/156) | 56.67% (17/30) | 60.75% (113/186) |
| Aztreonam | 94.12% (48/51) | 94.44% (136/144) | 95.51% (85/89) | 93.40% (99/106) | 93.94% (155/165) | 96.67% (29/30) | 94.36% (184/195) |
| Ampicillin/sulbactam | 72.00% (36/50) | 59.29% (83/140) | 59.09% (52/88) | 65.69% (67/102) | 63.41% (104/164) | 57.69% (15/26) | 62.63% (119/190) |
| Piperacillin | 49.02% (25/51) | 45.83% (66/144) | 46.07% (41/89) | 47.17% (50/106) | 47.88% (79/165) | 40.00% (12/30) | 46.67% (91/195) |
| Piperacillin/tazobactam | 82.35% (42/51) | 78.47% (113/144) | 80.90% (72/89) | 78.30% (83/106) | 79.39% (131/165) | 80.00% (24/30) | 79.49% (155/195) |
| Gentamicin | 78.43% (40/51) | 65.97% (95/144) | 66.29% (59/89) | 71.70% (76/106) | 68.48% (113/165) | 73.33% (22/30) | 69.23% (135/195) |
| Tetracycline | 61.70% (29/47) | 48.20% (67/139) | 48.81% (41/84) | 53.92% (55/102) | 51.92% (81/156) | 50.00% (15/30) | 51.61% (96/186) |
| Tigecycline | 100.00% (48/48) | 99.17% (120/121) | 100.00% (73/73) | 98.96% (95/96) | 99.30% (142/143) | 100.00% (26/26) | 99.41% (168/169) |
| Cefepime | 62.75% (32/51) | 59.03% (85/144) | 56.18% (50/89) | 63.21% (67/106) | 59.39% (98/165) | 63.33% (19/30) | 60.00% (117/195) |
| Cefuroxime | 60.00% (3/5) | 20.00% (1/5) | 0.00% (0/50) | 80.00% (4/5) | 40.00% (4/10) | - (0/0) | 40.00% (4/10) |
| Cefoperazone/sulbactam | 82.35% (42/51) | 76.92% (110/143) | 76.14% (67/88) | 80.19% (85/106) | 78.79% (130/165) | 75.86% (22/29) | 78.35% (152/194) |
| Cefotaxime | 57.45% (27/47) | 50.36% (70/139) | 48.81% (41/84) | 54.90% (56/102) | 51.28% (80/156) | 56.67% (17/30) | 52.15% (97/186) |
| Ceftazidime | 70.59% (36/51) | 65.28% (94/144) | 65.17% (58/89) | 67.92% (72/106) | 66.06% (109/165) | 70.00% (21/30) | 66.67% (130/195) |
| Ceftazidime/avibactam | 100.00% (51/51) | 99.29% (139/140) | 100.00% (86/86) | 99.05% (104/105) | 99.38% (161/162) | 100.00% (29/29) | 99.48% (190/191) |
| Cefazolin | 30.30% (10/33) | 25.51% (25/98) | 0.00% (0/50) | 43.21% (35/81) | 26.13% (29/111) | 30.00% (6/20) | 26.72% (35/131) |
| Imipenem | 82.35% (42/51) | 86.81% (125/144) | 84.27% (75/89) | 86.79% (92/106) | 85.98% (141/164) | 83.87% (26/31) | 85.64% (167/195) |
| Colistin | 97.87% (46/47) | 99.28% (138/139) | 100.00% (84/84) | 98.04% (100/102) | 98.72% (154/156) | 100.00% (30/30) | 98.92% (184/186) |
| Levofloxacin | 61.22% (30/49) | 51.06% (72/141) | 49.43% (43/87) | 57.28% (59/103) | 54.32% (88/162) | 50.00% (14/28) | 53.68% (102/190) |

**Supplementary table 7. Results of Enterococcus faecalis antimicrobial susceptibility testing.**

| Antibiotics | Stone-associated | Non-stone-associated | 2022 | 2023 | Outpatient clinic | Ward | Total |
| --- | --- | --- | --- | --- | --- | --- | --- |
| Ampicillin | 100.00% (139/139) | 100.00% (397/398) | 100.00% (238/238) | 99.67% (298/299) | 99.80% (491/492) | 100.00% (45/45) | 99.81% (536/537) |
| Nitrofurantoin | 98.56% (137/139) | 98.24% (391/398) | 97.90% (233/238) | 98.66% (295/299) | 98.17% (483/492) | 100.00% (45/45) | 98.32% (528/537) |
| Gentamicin (high level) | 78.03% (103/132) | 78.70% (313/385) | 72.80% (174/239) | 87.05% (242/278) | 79.87% (377/472) | 86.67% (39/45) | 80.46% (416/517) |
| Erythromycin | 5.04% (7/139) | 6.00% (24/400) | 5.86% (14/239) | 5.67% (17/300) | 6.28% (31/494) | 0.00% (0/45) | 5.75% (31/539) |
| Ciprofloxacin | 30.94% (43/139) | 34.75% (139/400) | 32.64% (78/239) | 34.67% (104/300) | 34.21% (169/494) | 28.89% (13/45) | 33.77% (182/539) |
| Linezolid | 100.00% (134/134) | 100.00% (386/386) | 100.00% (230/230) | 100.00% (290/290) | 100.00% (478/478) | 100.00% (42/42) | 100.00% (520/520) |
| Chloramphenicol | 63.50% (87/137) | 61.90% (247/399) | 61.44% (145/236) | 63.00% (189/300) | 62.73% (308/491) | 57.78% (26/45) | 62.31% (334/536) |
| Minocycline | 12.95% (18/139) | 13.07% (52/398) | 12.55% (30/239) | 13.42% (40/298) | 13.01% (64/492) | 13.33% (6/45) | 13.04% (70/537) |
| Penicillin G | 97.84% (136/139) | 95.50% (382/400) | 96.23% (230/239) | 96.00% (288/300) | 95.75% (473/494) | 100.00% (45/45) | 96.10% (518/539) |
| Teicoplanin | 100.00% (138/138) | 100.00% (400/400) | 100.00% (239/239) | 100.00% (299/299) | 100.00% (493/493) | 100.00% (45/45) | 100.00% (538/538) |
| Vancomycin | 94.96% (132/139) | 94.50% (378/400) | 94.98% (227/239) | 94.33% (283/300) | 94.74% (468/494) | 93.33% (42/45) | 94.62% (510/539) |
| Levofloxacin | 60.43% (84/139) | 62.75% (251/400) | 61.09% (146/239) | 63.00% (189/300) | 62.75% (310/494) | 55.56% (25/45) | 62.15% (335/539) |

**Supplementary table 8. Results of group B streptococcus antimicrobial susceptibility testing.**

| Antibiotics | Stone-associated | Non-stone-associated | 2022 | 2023 | Outpatient clinic | Ward | Total |
| --- | --- | --- | --- | --- | --- | --- | --- |
| Erythromycin | 16.92% (11/65) | 18.18% (22/121) | 17.50% (14/80) | 17.92% (19/106) | 15.34% (25/163) | 34.78% (8/23) | 17.74% (33/186) |
| Clindamycin | 18.75% (12/64) | 23.28% (27/116) | 20.25% (16/79) | 22.77% (23/101) | 20.75% (33/159) | 28.57% (6/21) | 21.67% (39/180) |
| Linezolid | 100.00% (65/65) | 100.00% (120/120) | 100.00% (79/79) | 100.00% (106/106) | 100.00% (163/163) | 100.00% (22/22) | 100.00% (185/185) |
| Penicillin G | 100.00% (64/64) | 100.00% (120/120) | 100.00% (80/80) | 100.00% (104/104) | 100.00% (161/161) | 100.00% (23/23) | 100.00% (184/184) |
| Ceftriaxone | 100.00% (65/65) | 100.00% (121/121) | 100.00% (80/80) | 100.00% (106/106) | 100.00% (163/163) | 100.00% (23/23) | 100.00% (186/186) |
| Vancomycin | 100.00% (65/65) | 100.00% (120/120) | 100.00% (80/80) | 100.00% (105/105) | 100.00% (163/163) | 100.00% (22/22) | 100.00% (185/185) |
| Levofloxacin | 52.31% (34/65) | 54.55% (66/121) | 52.50% (42/80) | 54.72% (58/106) | 54.60% (89/163) | 47.83% (11/23) | 53.76% (100/186) |
